# Estimating the number of unvaccinated Chinese workers against yellow fever in Angola

**DOI:** 10.1186/s12879-018-3084-y

**Published:** 2018-04-17

**Authors:** A. Wilder-Smith, E. Massad

**Affiliations:** 1 Lee Kong Chian School of Medicine, Nanyang Technological University, Singapore, Singapore; 2 Institute of Public Health, University of Heidelberg, Heidelberg, Germany; 3 London School of Tropical Medicine and Hygiene, London, UK; 4 School of Medicine, University of Sao Paulo and LIM 01 HCFMUSP, Sao Paulo, Brazil; 5 501100007227 School of Applied Mathematics, Fundacao Getulio Vargas, Rio de Janeiro, Brazil

**Keywords:** Yellow fever, International health regulations, Air travel, Importation, Proof of yellow fever vaccination, Angola, Chinese

## Abstract

**Background:**

A yellow fever epidemic occurred in Angola in 2016 with 884 laboratory confirmed cases and 373 deaths. Eleven unvaccinated Chinese nationals working in Angola were also infected and imported the disease to China, thereby presenting the first importation of yellow fever into Asia. In Angola, there are about 259,000 Chinese foreign workers. The fact that 11 unvaccinated Chinese workers acquired yellow fever suggests that many more Chinese workers in Angola were not vaccinated.

**Methods:**

We applied a previously developed model to back-calculate the number of unvaccinated Chinese workers in Angola in order to determine the extent of lack of vaccine coverage.

**Results:**

Our models suggest that none of the 259,000 Chinese had been vaccinated, although yellow fever vaccination is mandated by the International Health Regulations.

**Conclusion:**

Governments around the world including China need to ensure that their citizens obtain YF vaccination when traveling to countries where such vaccines are required in order to prevent the international spread of yellow fever.

**Electronic supplementary material:**

The online version of this article (10.1186/s12879-018-3084-y) contains supplementary material, which is available to authorized users.

## Background

A yellow fever (YF) epidemic began in Angola, in December 2015 in the capital Luanda, and by September 2016, a total of 884 laboratory confirmed cases had been reported with 373 deaths [1]. The national spread of YF was characterized by fast exponential growth (doubling time of 5–7 days) and fast spatial expansion from Luanda, the capital of Angola [2]. YF spread via trade and travel routes within the African continent to Kenya and the Democratic Republic of the Congo [3]. Of even greater concern was that for the first time in known history, laboratory-documented YF was exported from Africa to China via returning travelers thus putting a large proportion of highly populous China at risk because the mosquito vector (*Aedes aegypti*) is present in more than half of the country [4–6]. In total, there were eleven unvaccinated Chinese foreign workers working in Angola who were infected during the 2016 YF outbreak in Angola and diagnosed at the time when they returned to China. More Chinese may have been infected in Angola who remained in Angola, but no documentation on such cases is available.

In Angola, there are about 259,000 Chinese foreign workers, the second largest contingent of Chinese in the continent of Africa after South Africa [http://www.who.int/csr/resources/publications/dengue/en/012-23.pdf]. According to the International Health Regulations (IHR), proof of vaccination is required for entry into China from certain YF endemic countries, including Angola [7].

The fact that 11 Chinese workers acquired yellow fever due to lack of prior vaccination points suggests that many more Chinese workers in Angola were not vaccinated. We hence attempted to back-calculate the number of unvaccinated Chinese workers in Angola in order to determine the extent of lack of vaccine coverage.

## Methods

We applied a previously developed mathematical model [8] and used the following figures for the parameters: a population size for Angola of 25,021,974 [9], 884 confirmed YFV cases in Angola between December 2015 and July 2016 (30 weeks), and 259,000 Chinese foreign workers in Angola [http://www.who.int/csr/resources/publications/dengue/en/012-23.pdf], eleven of whom acquired symptomatic laboratory-confirmed yellow fever.

**Fig. 1:**
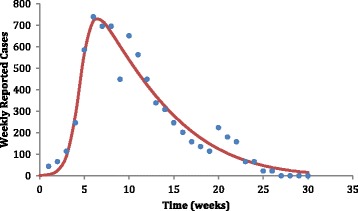
Fitting a continuous function (line) to actual incidence of weekly reported new infections of YF in Angola, 2016 (Mean Square Error = 52.9767). The fitting parameters were *c*_1_ = 7.3778 ×10^−5^, *c*_2_ = -8.61647, *c*_3_ = 325.023 *c*_4_ = 1.45759 and *c*_5_ = 4.60653

We first calculated the force of infection in Angola based on the epidemic curve of the outbreak from December 2015 until July 2016. Our model is a Ross-Macdonald model for vector-borne infections; its structure is of a stochastic SIR kind of model to take account for the high mortality observed in serious cases of YF. From the epidemic curve we calculated the local YF force of infection, *λ*(*t*). This is done by, firstly, fitting a continuous function to the actual incidence, *Inc*(*t*), of new infections (assuming that the 884 reported cases represented only 12% of all infections, that is, an asymptomatic-to-symptomatic ratio of 8.8:1 [10], which had the ‘Gaussian’ form:

$$ Inc(t)={c}_1\exp \left[-\frac{{\left(t-{c}_2\right)}^2}{c_3}\right]\left[\frac{1}{1+\exp \left(-{c}_4\left(t-{c}_5\right)\right)}\right] $$

where *c*_1_is a scale parameter that determines the maximum incidence, *c*_2_ is the age with the maximum incidence, *c*_3_ represents the width of the time-dependent incidence function, and *c*_4_ and. *c*_5_ are fitting parameters with no physical meaning. The fitting accuracy can be observed in Fig. 1. From the *Inc*(*t*) function, we calculated, through the Ross-Macdonald SIR model, the local YF force of infection, *λ*(*t*) which is related to the risk of at least one infection, *π*(*t*), using the following equation [8]:

$$ \pi (t)=\frac{\underset{0}{\overset{t}{\int }}\lambda \left({t}^{\hbox{'}}\right){dt}^{\hbox{'}}}{\underset{0}{\overset{t}{\int }}\left[\lambda \left({t}^{\hbox{'}}\right)-\gamma \right]{dt}^{\hbox{'}}}\left\{\exp \left(-\gamma t\right)-\exp \left[-\underset{0}{\overset{t}{\int }}\lambda \left({t}^{\hbox{'}}\right){dt}^{\hbox{'}}\right]\right\} $$

where *γ* is a ‘removal’ rate composed by the natural mortality rate, the recovery rate from infection and the additional mortality due to the disease. The removal rate was estimated considering a viraemic period of 5 days (1.36*10^− 1^ weeks^− 1^), a hosts’ life expectancy of 65 years (2.96*10^− 4^ weeks^− 1^) and a lethality of yellow fever of 41% in 4 weeks (7.14*10^− 1^ weeks^− 1^).

## Results

The cumulative probability of at least one symptomatic case in the period of 30 weeks (that is, the sum of all the 30 weekly probabilities of at least one symptomatic case during the outbreak) is 3.53*10^− 4^. The total number of cases retrieved by the model was 842 symptomatic cases among the Angolese populations and 10 symptomatic cases among the Chinese cohort, which is consistent with the actual numbers. To explain the observed 11 symptomatic cases among the Chinese foreign workers in Angola (which implies in 97 actual infections), we calculated that all the 259,000 Chinese must have been susceptible, that is, non-vaccinated. In other words, according to these calculations, none of the Chinese workers had received the YF vaccine. There may be different reasons for the absence of vaccination. For example, for one case there were medical contraindications with regards to yellow fever vaccination [11].

## Discussion

Yellow fever, like any vector-borne infection, may be focal in space and, therefore, some of the cases may have clustered around hot-spots of transmission which could result in non-negligible heterogeneities. This would imply that Chinese workers were submitted to different forces of infections compared to the endemic population, for instance, by living/working in different places with different mosquitoes densities. The risk would then have the equation:

$$ \pi \left(t,s\right)=\frac{\underset{0}{\overset{t}{\int }}\underset{0}{\overset{s}{\int }}\lambda \left({t}^{\hbox{'}},{s}^{\hbox{'}}\right){dt}^{\hbox{'}}{ds}^{\hbox{'}}}{\underset{0}{\overset{t}{\int }}\underset{0}{\overset{s}{\int }}\left[\lambda \left({t}^{\hbox{'}},{s}^{\hbox{'}}\right)-\gamma \right]{dt}^{\hbox{'}}{ds}^{\hbox{'}}}\left\{\exp \left(-\gamma t\right)-\exp \left[-\underset{0}{\overset{t}{\int }}\underset{0}{\overset{s}{\int }}\lambda \left({t}^{\hbox{'}},{s}^{\hbox{'}}\right){dt}^{\hbox{'}}{ds}^{\hbox{'}}\right]\right\} $$

where *s* denotes the points in space.

However, the lack of information on the spatial distribution of the cases among Chinese workers did not allow us to refine the model to include such spatial heterogeneities. Another reason why we did not consider the inevitable uncertainties involved in our estimations was that they would not have had any impact on the final conclusion of the model. We only included the 11 confirmed cases picked up in China, but the overall number of yellow fever cases in Chinese workers was likely higher as many would not have travelled back to China in that time period. In other words, even with as few as 11 cases, our models backcalculate that 100% of the 250,000 workers were unvaccinated at the time of the yellow fever outbreak in Angola. A higher number of infected workers would not change the result of 100%. There is also some uncertainty about the actual nunber of Chinese workers living in Angola at the time of the outbreak late 2015 to mid 2016, with the Angola-China Industrial and Commerce Association stating that by 2017 the overall number had fallen to about a quarter of what it was in 2013–2014 (https://qz.com/940619/chinese-traders-changed-south-africa-now-theyre-leaving/). However, for 2016 the numbers were in the order of magnitude that we used for our calculation according to two other sources (http://www.china-invests.net/20160614/40671.aspx) (https://macaudailytimes.com.mo/brazil-angola-top-five-countries-chinese-investment-south-atlantic.html). Even if the overall Chinese population was smaller than we assumed for our models, this would only even more reinforce that 100% of the Chinese population in Angola at the time of the outbreak was unvaccinated.

In the Additional file 1 we show how to include the uncertainties involved in the process of modeling.

Our calculations unmask a serious problem. Despite the fact that YFV vaccination is mandated for Angola by the International Health Regulations (IHR) [12], not only the 11 travelers from Angola to China were documented to be unvaccinated, but also the vast majority if not all of the Chinese residing in Angola at the time were unvaccinated based on our modelling. The Chinese government immediately responded to the crisis by employing medical teams to Angola to vaccinate the Chinese population in that country [13].

China has been dominating global outbound travel for the past decade, especially after achieving double-digit growth in tourism expenditures every year since 2004. The total number of outbound travellers from China rose by 11 million from 2014 to reach 128 million in 2015 [14]. A recent study estimated that the annual travel volume of Chinese travelers out of China into YF endemic countries was 466,832, the majority of which traveled to Nigeria (*n* = 227,694), Angola (*n* = 69,334) and Brazil (*n* = 65,954). Such data are important for demand forecasting of yellow fever vaccines for Chinese travelers, as China has its own yellow fever vaccine manufacturer (http://www.dcvmn.org/_China-National-Biotec-Group-Company-Limited_).

## Conclusion

Governments around the world including China need to ensure that their citizens obtain YF vaccination when traveling to countries where such vaccines are recommended and/or required according to IHR, in order to prevent the rapid international (and long-haul) spread of YF as it happened in 2016. Tightening border controls is essential to ensure that incoming travelers from YF endemic countries into vulnerable countries (as defined by IHR) carry proof of YF vaccination.

## Additional file

Additional file 1: Appendix-Uncertainties in the model. (DOCX 21 kb)

## Abbreviations

IHR: International Health Regulations
SIR: Susceptible-Infected-Recovered
YF: Yellow Fever

## References

[1] World Health Organization (WHO). http://www.afro.who.int/en/yellow-fever/sitreps/item/9085-situation-report-yellow-fever-outbreak-in-angola-29-september-2016.html (accessed 30 Oct 2016).

[2] Kraemer MU, Faria NR, Reiner RC (2017). Spread of yellow fever virus outbreak in Angola and the Democratic Republic of the Congo 2015-16: a modelling study. Lancet Infect Dis.

[3] Wilder-Smith A, Monath TP. Responding to the threat of urban yellow fever outbreaks. Lancet Infect Dis. 2017;17(3):248-50. 10.1016/S1473-3099(16)30588-6 28017560

[4] Wu F, Liu Q, Lu L, Wang J, Song X, Ren D (2011). Distribution of Aedes albopictus (Diptera: Culicidae) in northwestern China. Vector Borne Zoonotic Dis.

[5] Wang G, Zhang H, Cao X (2014). Using GARP to predict the range of Aedes aegypti in China. Southeast Asian J Trop Med Public Health.

[6] Kraemer MU, Sinka ME, Duda KA (2015). The global compendium of Aedes aegypti and ae. Albopictus occurrence. Scientific data.

[7] Hardiman M, Wilder-Smith A (2007). The revised international health regulations and their relevance to travel medicine. J Travel Med.

[8] Lopez LF, Amaku M, Coutinho FA (2016). Modeling importations and exportations of infectious diseases via travelers. Bull Math Biol.

[9] The World Bank. World Development Indicators 22 Nov 2016 2015. http://databank.worldbank.org/data/reports.aspx?source=2&type=metadata&series=SP.POP.TOTL. Accessed Mar 2017.

[10] Johansson MA, Vasconcelos PF, Staples JE (2014). The whole iceberg: estimating the incidence of yellow fever virus infection from the number of severe cases. Trans R Soc Trop Med Hyg.

[11] Ling Y, Chen J, Huang Q (2016). Yellow fever in a worker returning to China from Angola, march 2016. Emerg Infect Dis.

[12] Wilder-Smith A, Gubler DJ, Weaver SC, Monath TP, Heymann DL, Scott TW (2017). Epidemic arboviral diseases: priorities for research and public health. Lancet Infect Dis.

[13] Yellow fever-China. 2016. http://www.who.int/csr/don/22-april-2016-yellow-fever-china/en/. Accessed Mar 2017.

[14] Glaesser D, Kester J, Paulose H, Alizadeh A, Valentin B. Global travel patterns: an overview. J Travel Med. 2017;24(4). 10.1093/jtm/tax007. 10.1093/jtm/tax007 28637267

