# Supplementary material for: Estimating the number of unvaccinated Chinese workers against yellow fever in Angola
Source: BMC Infect Dis. 2018 Apr 17;18:185. doi: 10.1186/s12879-018-3084-y (PMC5905133; doi:10.1186/s12879-018-3084-y)
Supplement: Supplementary file 1 — Appendix-Uncertainties in the model. (DOCX 21 kb) [file 12879_2018_3084_MOESM1_ESM.docx]

**Additional Files**

**Additional File 1:** Appendix - Uncertainties in the model

Here we show how to include the uncertainties involved in the process of modeling the risk of yellow fever.

First we calculate the variance of the probability distribution ($\sigma^{2}$) for the number of infected individuals at time t estimated from the Probability Generating Function $G(z,t)$ as follows:

$\begin{matrix} \sigma_{Risk}^{2}=\pi(t)\left[1-\pi(t)\right]=\left\{\left[\frac{\int_{0}^{t} \lambda(s)ds}{\int_{0}^{t} \left[\lambda(s)-\gamma\right]ds}\left\{\exp(-\gamma t)-\exp\left[-\int_{0}^{t} \lambda(s)ds\right]\right\}\right]\times\right. \\ \left.\left[1-\frac{\int_{0}^{t} \lambda(s)ds}{\int_{0}^{t} \left[\lambda(s)-\gamma\right]ds}\left\{\exp(-\gamma t)-\exp\left[-\int_{0}^{t} \lambda(s)ds\right]\right\}\right]\right\} \end{matrix}$ (A1)

Therefore, assuming a normal distribution in the errors, the risk of yellow fever acquisition at time *t, or Risk*(*t*), may be estimated using the following equation:

$Risk(t)=\pi(x)\pm1.96\sqrt{\frac{\sigma_{Risk}^{2}}{N}}$ (A2)

where the second term is the 95% confidence interval. In addition, we also considered the error propagation derived from the fitting procedures of the force of infection, $\sigma_{\lambda}(t)$which may be estimated as follows:

(A3)
